# A Review of Bonding Immiscible Mg/Steel Dissimilar Metals

**DOI:** 10.3390/ma11122515

**Published:** 2018-12-11

**Authors:** Gang Song, Taotao Li, Jingwei Yu, Liming Liu

**Affiliations:** Key Laboratory of Liaoning Advanced Welding and Joining Technology, School of Material Science and Engineering, Dalian University of Technology, Dalian 116024, China; songgang@dlut.edu.cn (G.S.); yujw@mail.dlut.edu.cn (J.Y.); liulm@dlut.edu.cn (L.L.)

**Keywords:** Mg alloy, steel, welding, Mg/steel interface, mechanical property

## Abstract

The challenge of joining immiscible Mg/Steel dissimilar metals lies in the absence of an Fe-Mg intermetallic or Fe-Mg solid solution in an Mg-Fe system, and differences in their physical and chemical properties. Promoting interfacial reaction and regulating the composition of interface layer are beneficial for the formation of an Mg/steel interface layer and to obtain an effective Mg/steel joint. This research work focusses on the bonding of immiscible Mg/steel dissimilar metals: First, an Mg/steel interface layer was designed by controlling the composition of added alloy elements and trace elements in the base metal. Second, the Mg/steel dissimilar metals welding methods were divided into three parts—solid state welding, welding-brazing and fusion welding. The main distinctions between them were difference in interfacial temperature, thickness of interface layer, and type of compound. Third, the orientation relationships (OR) of the Mg/interface layer system and the interface layer/steel system was investigated. In this review, the effect of welding processing parameters, addition of alloy elements, base metal, and different welding methods on the joint’s performance was studied. The mechanical property, microstructure, interface layer and metallurgical reactions of the joint were also examined. The most recent progress in joining immiscible Mg/Steel dissimilar metals and future research prospects are presented at the end of the paper.

## 1. Introduction

### 1.1. Background

Mg alloy has gradually become a competitive new light structural material. It is now being widely used in the fields of electronic communication, automobile, and aerospace. Steel materials will continue to occupy a dominant position in the application of industrial production with the successful development of high-strength steel. Some steel structures were possibly replaced by lightweight metals because of the rapid increase of lightweight structural materials. The application of lightweight materials in the automotive industry has become common. Automotive companies are using lightweight materials to create better and more-developed automobiles. Dissimilar metal hybrid structures combine the advantages of two metals (such as Al/steel [1], Al/Mg [2], Al/Cu [3], Al/Ti [4], Ti/steel [5,6] and Mg/steel hybrid structures [7], etc), and can achieve the purpose of being lightweight while satisfying operating requirements.

### 1.2. The Challenge of Mg/Steel Welding

The physical and chemical properties of Mg and Fe are shown in Table 1. The huge differences between Mg and Fe lead to the following challenges for Mg/steel joints.

**Figure 1:**
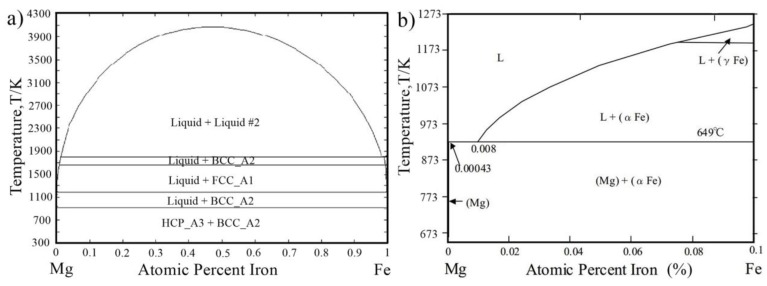
Mg-Fe binary phase diagram [9].

(1) Mg/steel joint welding was not similar to the Al/steel and Al/Mg joint. A large number of Fe-Al compounds are detected in the Al/Fe joint, which influence the Al/Fe joint performance. However, there was no intermetallic compound (IMC) forming in the Mg/Fe joint, and little solid solubility in the Mg-Fe system (Figure 1). Therefore, it was difficult to form an interface layer and obtain an effective Mg/steel joint.

(2) The melting point of Fe (about 1811 K) is much higher than the boiling point of Mg (1363 K). Mg is directly evaporated as steel melting. The thermal expansion coefficient of Mg is more than twice as big as that of Fe. For these reasons, heat input and welding distortion are particularly important during the Mg/steel welding process.

### 1.3. Research Status and Progress

The challenges of bonding immiscible Mg/Steel dissimilar metals are no Fe-Mg IMCs and little solid solubility in the Mg-Fe system, and obvious differences in the physical and chemical properties of Mg and Fe. Hence, knowing how to obtain an interface layer with certain thickness is the key to the bonding of immiscible Mg/Steel dissimilar metals. Promoting interfacial reaction could form a Mg/steel interface layer and obtain an effective Mg/steel joint. At present, the research on joining immiscible Mg/steel dissimilar metals mainly focuses on the following aspects. First, the elements of the interface layer mainly come from the base metal or by adding alloy elements. Therefore, the purpose of designing different types of interface layers could be achieved by choosing different components of the base metal and suitable alloy elements, such as, Mg base filler wires (AZ31, AZ61 and AZ91) and interlayer (Al, Cu, Ni, Cu-Zn foil layers or Ni, Zn, Sn coatings, et al.). The alloy elements could form IMCs or solid solutions with Mg and Fe may be used as a criterion, such as Al, Cu, Ni, Zn, Cu, Sn, not Ti and V (no IMCs or solid solutions with Mg), etc. Secondly, the change of interfacial temperature has an important influence on the type and thickness of the interface layer, such as Mg-Zn low melting point eutectic being detected near the Mg/steel interface during solid state welding, the melting point of IMC (Al_8_Mn_5_) nearly Mg boiling point being detected near the Mg/steel interface during welding-razing, and the high melting point phase (AlNi) detected near the Mg/steel interface during fusion welding. Lastly, the interface layers were mainly composed of IMCs. Different IMCs exist in the Mg/steel interface, which exhibited different mechanical properties. An edge-to-edge matching model is used for predicting orientation relationships (OR) of Mg/IMC and IMC/steel, which could characterize interface bonding strength. Based on the above ideas, design and control of the composition of interface layer could obtain a better Mg/steel joint. No matter which way was adopted to weld Mg alloy and steel, a better Mg/steel joints could be obtained, and some techniques were found to be very promising. A detailed discussion on the research of bonding immiscible Mg/steel dissimilar metals is presented in the following section.

## 2. Solid State Processes

### 2.1. Friction Stir Welding

Friction stir welding (FSW) produces heat between the pin and the shoulder, which melts the plastic in the workpiece. The stir head rotates, mixes, and moves in the welding direction. At the same time, a metallurgical reaction takes place at the interface of dissimilar metals. FSW ensures that the joint has no related melting welding defects, and thermal deformation and residual stress is small. Immiscible Mg/steel dissimilar metals can be bonded by this technology. The Zn coating is key to obtaining effective joints during most Mg/steel friction stir welding [10,11,12,13,14]. The purpose of Zn coating is to remove the oxide film and squeeze the new Zn phase to the edge, which could improve the weldability of Mg/steel.

**Figure 2:**
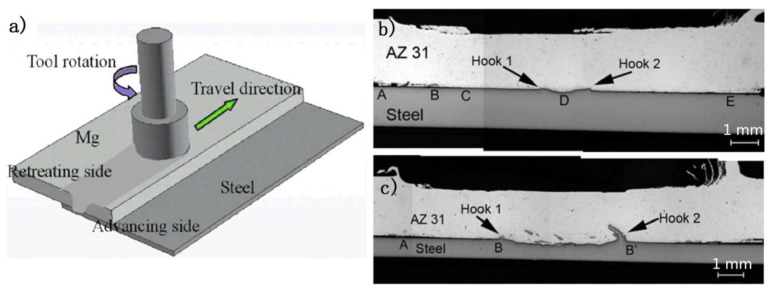
Friction stir lap welding process [15] (**a**) schematic illustrations; (**b**,**c**) cross-sectional macrograph of AZ31 to 1.5 mm and 0.8 mm steel weld.

**Figure 3:**
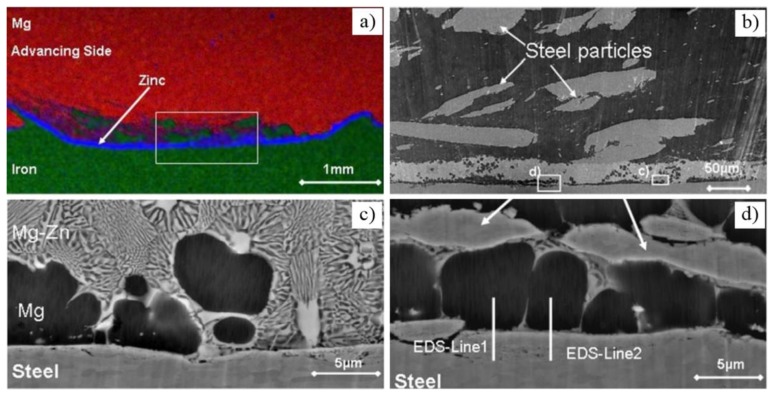
FSW Mg/Zn coated steel process [20] (**a**) EDX map of cross-section; (**b**) stir zone near Mg/steel interface; (**c**,**d**) enlarged drawing in (**b**).

Figure 2 shows the typical Mg/steel lap joint by FSW [15]; it is seen that the process stirs some steel particles in the Mg alloy stir zone [16,17,18,19]. The bonding mechanism of Mg/steel is mainly divided into two types: First, the Mg-Zn low melting point eutectic formed near the Mg/steel interface by the intervention of Zn coating, as shown in Figure 3 [20]. The forge force could obtain 8.2 kN at a higher welding speed, which was higher than that of the lower welding speed. This study showed that the forced material flow by stirring (not welding speed) was the main reason why the liquid layer flows out of the interface.

**Figure 4:**
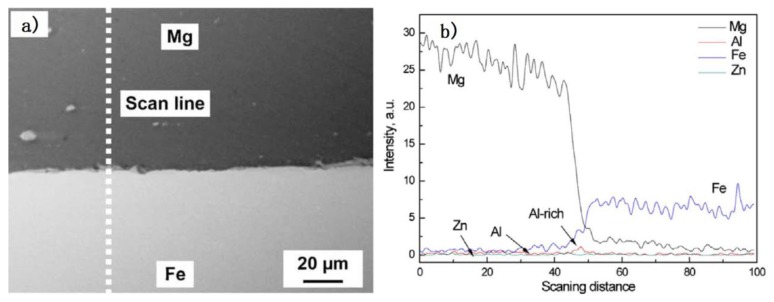
FSW Mg/Zn coated steel process [17] (**a**,**b**) SEM micrographs near Mg/steel interface and EDS analysis results along the scan line in (**a**).

Second, the liquid reaction products were extruded far from the center of the weld, spreading along the interface and then piling into the joining interface [14]. Low melting point eutectic was squeezed out of the Mg/steel interface, clearing the steel surface. This helps promote mutual diffusion in Mg alloy/steel layers. The Mg-Zn layer was formed at the side of the mechanically bonded region, as shown in Figure 4. This increased width of the mechanically bonded region may be responsible for producing joint strengths close to the parent material strength. Since the FSW tool vigorously stirred and fragmented the bottom of the electro–galvanized steel sheet welding joint, there was no opportunity for the molten Zn coating to flow laterally at several locations. As a result, a Zn-Mg solidified phase formed at those regions. The results of Mg, Zn, Al and Fe linear SEM-EDS is shown in Figure 4b. The Al element was enriched in the Mg/steel interface, and the Zn element changed little along the EDS analysis results. The Mg/steel lap joints failed at the Mg/steel interface. The common IMCs at the Mg/steel interface were: Fe_4_Al_13_ and Fe_3_Al [13,17,18,19,21], Fe_2_Al_5_ [10] and FeAl_2_ [12]. These results show that the intervention of Zn improved the interface bonding state of Mg/steel lap joints.

**Figure 5:**
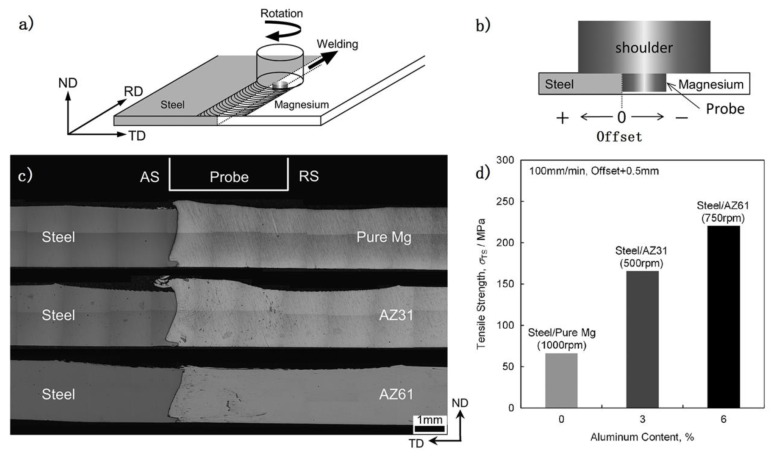
FSW Mg/steel butt joint process [22] (**a**) schematic illustrations; (**b**) tool offset position; (**c**) OM images of the interfaces on the cross section; (**d**) relationship between the optimal tensile strength of the joint and Al content.

FSW technical was also adopted to weld a Mg/steel butt joint. Kasai et al. [22] studied the effect of Al content in the Mg alloy on the mechanical properties of steel/Mg butt joint by FSW. From Figure 5, it is evident that maximum tensile strength appeared in the AZ61/steel butt joint. The higher the Al content (pure Mg, AZ31 and AZ61 Mg alloy), the greater the tensile strength of the joint was. The Al elements played a key role in bonding Mg/steel dissimilar metals [23]. The Fe-Al IMC (Fe_2_Al_5_) layer was formed at the Mg/steel interface and primarily detected in AZ series Mg alloy/steel joints. Besides, the thickness of the interface layer also had a great influence on the strength of joints. The thinner the interface layer, the greater strength of the joint.

By optimizing the welding parameters to obtain excellent Mg/steel FSW joints, the maximum shear load of 2.9 kN was obtained by AM60/galvanized DP600 steel friction stir spot welding (FSSW) joints [24]. The shear load of AZ31/galvanized Q235 steel FSSW joint reached 4.3 kN with pre-existing Fe_2_Al_5_ on the surface of the steel. ZEK100 Mg alloy and Zn coated DP600 steel was welded by refill FSSW and obtained 4.7 kN shear load [12]. AZ31B/galvanized mild steel joints were welded by friction stir keyholeless spot welding and obtained maximum shear strength of 8.7 kN [13]; the AZ31B/DP600 galvanized steel joints reached 8.7 kN [25] and 10.36 kN [11]. AZ31 Mg alloy and low carbon Zn coated steel joints obtained maximum failure loads of 2.3 kN [19] and 3.4 kN (65% of steel base metal) [18] by friction stir lap welding. The maximum shear strength of the AZ31/1.5 mm hot–dipped galvanized steel joints was found to be 7.07 kN [15]. AZ31B/Zn coated ultralow carbon steel lap joints could reach 8.2 kN for a higher welding speed [20]. In summary, there were several factors affecting the performance of the Mg/steel joint; they included a suitable IMC preset on the steel surface, adoption of friction stir keyholeless welding technology, and choice of Zn-coated steel.

**Figure 6:**
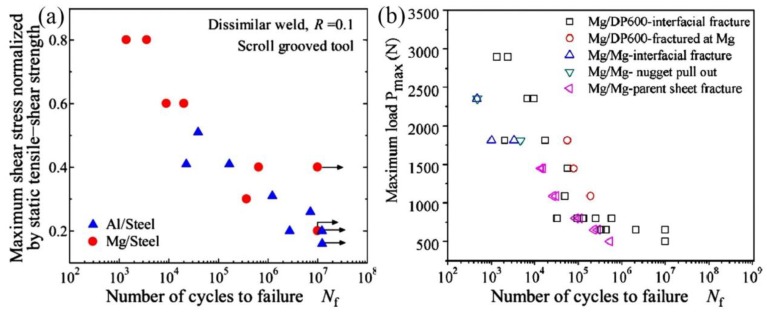
Relationship between maximum load and number of cycles to failure (**a**) Mg alloy/steel friction stir spot welds [26]; (**b**) Mg/steel dissimilar refill friction stir spot welds [14].

In addition, the fatigue behavior of Mg/steel friction stir spot welds was also studied [14,26], as shown in Figure 6. AZ31/low carbon steel sheets dissimilar metals were successfully welded by FSSW without a probe, and the tensile-shear fatigue strength of Mg/steel joint was comparable to Mg/Mg welds [26]. ZEK 100 Mg alloy/galvanized DP600 steel sheets was welded by refill FSSW [14]. Most of the fatigue fractures were broken at the Mg/steel interface, except stress 1087 N ≤ *P*_max_ ≤ 1812 N, whose fatigue fractures occurred at the Mg alloy base metal.

### 2.2. Diffusion Bonding

Diffusion bonding is a typical solid state technique suitable for joining Mg/steel dissimilar metals. The diffusion welding of Mg/steel is mainly based on the lap joint. It aims to close the workpiece surface at a high temperature and under a certain pressure, and to maintain a period of time to realize the mutual diffusion between the atoms in the workpiece surface. In the bonding of some dissimilar metals with little mutual solubility, the method of adding interlayers can be used to obtain a reliable and effective bonding. The advantage of this welding method is that welding temperature is low, and dissimilar materials can be bonded without damage to the properties of the base metal. Besides, welding deformation is small, and joint composition and organization is uniform. The determined influence on shear load of the joints was bonding time and temperature, followed by surface roughness and bonding pressure. There is no IMC between Mg/steel. Therefore, an interlayer should be added to achieve effective bonding between them.

As mentioned above, the interlayer is used as a bridge to promote the metallurgical bonding of Mg/steel joint. Ni and Cu were primary selected as the interlayer to join Mg alloy and steel, because they offer metallurgical compatibility with Mg and Fe. Different microstructures and bonded interface layer are formed under different interlayers.

**Figure 7:**
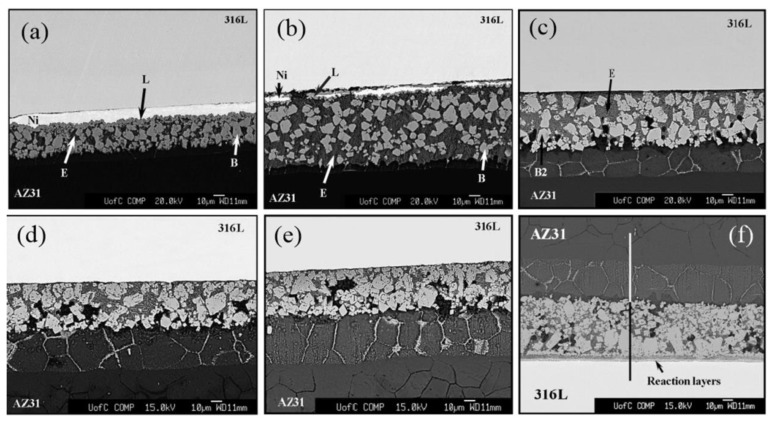
SEM micrographs of AZ31/Ni/316 L joint at 510 °C with different bonding time [27] (**a**) 3 min; (**b**) 5 min; (**c**) 10 min; (**d**) 20 min; (**e**) 30 min; (**f**) double stage bonding of 316 L/Ni/AZ31 with 20 min bonding time.

Elthalabawy et al. [27,28] used Ni interlayer and studied the microstructural evolution of double stage diffusion-brazed 316 L austenitic stainless steel to AZ31 Mg alloy. The effect of bonding times on joint microstructural evolution is shown in Figure 7a–e. A maximum shear strength of 46 MPa was obtained at 510 °C with 20 min bonding time, because of a eutectic phase surrounding the intermetallic B2 particles. To further improve the shear strength of Mg/steel joint, the double stage bonding of 316 L/Ni/AZ31 was adopted [27]. A solid-state Ni-Fe reaction layer preexisted on the steel surface. The Ni-Fe reaction layer on the steel surface bonds the interfacial structure after diffusion, instead of the Fe. The experimental results showed that the joint region reveals a thin reaction layer representing the Mg_2_Ni IMC and Mg-Ni eutectic phase (E). The maximum value of joint shear strength was 54 MPa by double stage diffusion brazing owing to the solid-state Ni-Fe reaction layer.

**Figure 8:**
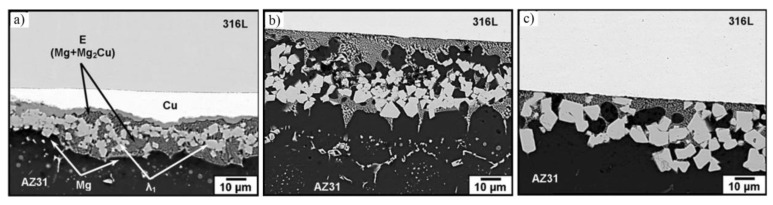
SEM micrographs of AZ31/Cu/316 L joint at 530 °C with different bonding time [29] (**a**) 5 min; (**b**) 15 min; (**c**) 30 min.

A pure Cu interlayer was also studied in the microstructural characteristics of AZ31/Cu/316 L joint by diffusion-brazing [29,30]. The effect of bonding time on microstructural evolution and bond shear strength were studied at 530 °C. The bonding process took place through a sequential occurrence of solid state diffusion of Cu into the Mg alloy, eutectic phase formation, interlayer dissolution, and isothermal solidification. The λ_1_ (Mg-Cu-Al) ternary intermetallic phase formed within the joint and concentrated into the center of the bond during the solidification stage is shown in Figure 8. The packing of the intermetallic (λ_1_) had a significant effect on the change of bond shear strength. The maximum shear strength 57 MPa was obtained at 510 °C with 20 min bonding time.

The addition of interlayer diffusion to Mg alloys and steel can solve the problems of Mg/steel immiscibility and lack of intermetallic compounds. Diffusion bonding can control weld microstructures precisely by setting the temperature, pressure, and heating time. These researches provide a feasible way to improve the properties of joints by promoting the Mg/steel metallurgical bonding adding interlayer.

## 3. Welding-Brazing

Welding-brazing utilizes the difference in melting point between the two alloys, which applies a heat source to the low melting point materials (base metal or filler material). After the low melting point Mg alloy is melted, the high melting point steel infiltrated, and a diffusion interface layer formed at the Mg/steel interface. Research studies on laser welding, arc welding, and laser-tungsten inert gas (TIG) hybrid welding have been carried out on the Mg/steel welding-brazing joint.

### 3.1. Laser Welding-Brazing

#### 3.1.1. Laser Welding without Any Filler Wire or Interlayer and Coatings

Laser self-welding only uses a laser source without filling interlayers and filler metals. In one case, the laser melted the Mg alloy, which wets and spreads on the surface of the steel, and forms a brazing joint [31,32,33,34]. In another instance, the laser was focused on the top surface of the steel plate and melted the Mg alloy by thermal conduction heat, thereby forming a brazed joint [35].

**Figure 9:**
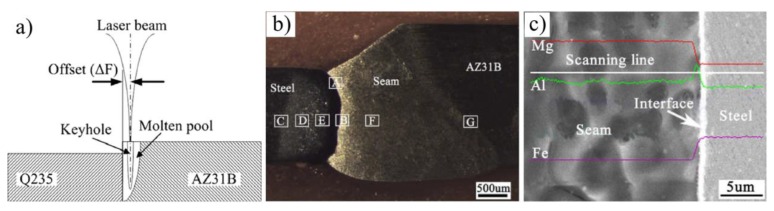
Laser penetration welding-brazing [32] (**a**) schematic diagram; (**b**) cross-section of butt joint; (**c**) interface characteristics of the lower part of joint and element distributions.

Firstly, laser-brazed AZ31 Mg alloy to Q235 steel joint was investigated by Miao et al. [31,32,33]. The Mg alloy was melted and the steel melting was restricted. The molten Mg alloy, as a filler metal, formed a successful welding brazing joint, as shown in Figure 9. A Mg/Fe transition layer was formed for the strong interfacial metallurgical reaction. The compounds were identified as Al-rich phases, such as Mg_17_Al_12_, Mg_2_Al_3_, FeAl and Fe_4_Al_13_ [33]. At the upper part of the joint, an obvious transition zone was found on the AZ31/Q235 interface, and the element distributions varied slowly in the interfacial layer, which was related to the little melting or dissolving of steel. At the lower parts, the transition zone was obscured and the elemental content changed abruptly due to the lower temperature, as shown in Figure 9e. The joint failed at the interface, and the average tensile strength of butt joints reached 185 MPa at 0.6 mm laser offset [32]. Jiang et al. also adopted laser welding-brazing to join AZ31B Mg alloy and AISI304 stainless steel [34]. The maximum joint strength was 211 MPa (89.8% Mg base metal) with the laser offset 0.2 mm to Mg alloy side. The interface reaction layer may be composed of Mg_17_Al_12_ and Mg_2_Ni because the fracture surface at steel side detected the two IMCs.

**Figure 10:**
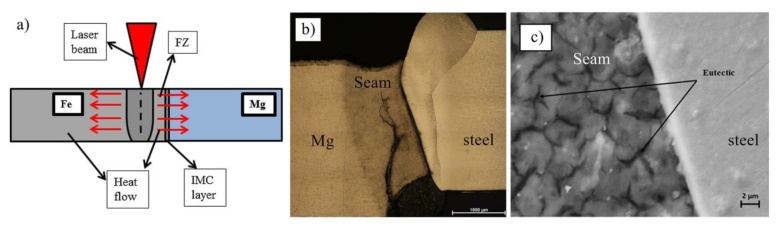
Laser offset welding of the AZ31/316 joint with 0.4 mm offset [35] (**a**) schematic diagram; (**b**) cross section of joint; (**c**) the microstructure near the Mg/steel interface.

Casalino et al. [35] put forward the laser offset to 316 stainless steel sides to bond with AZ31B/316, not using any interlayer or groove preparation, as showed in Figure 10. The joints could be bonded in the 0.3 mm and 0.4 mm offset conditions, and no bond for 0.5 mm offset. The ultimate tensile strength was close to 100 MPa. This technique provided a new idea that dissimilar metal welds with huge difference in physical parameters could be bonded with good tensile strength.

#### 3.1.2. Laser Welding with Filler Wire or Interlayer and Coatings

On the basis of laser self-welding, the interface metallurgical bonding can be promoted by filling the welding wire to make up the burning loss problem or by adding interlayers and coatings. These filler wires, interlayers and coatings included Zn [7,36,37,38,39,40], Ni [41,42,43], Al [44], Sn [45], AZ31 filler wire [7,37,39,46,47,48], AZ92 filler wire [36,42,43,49], and others alloy elements [38,49,50].

**Figure 11:**
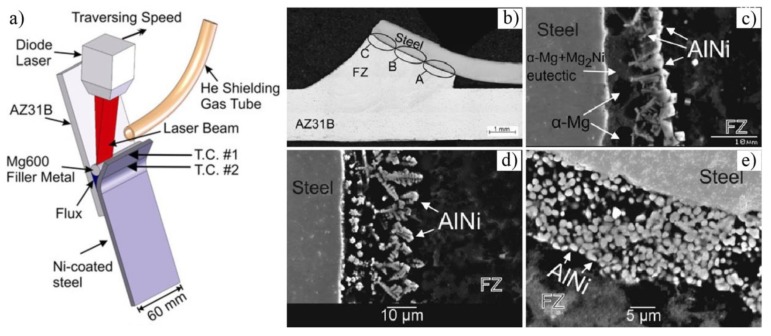
Laser welding of Mg/steel with Ni coating [43] (**a**) schematic diagram of laser bonding of AZ31 Mg and Ni electro-plated steel; (**b**) cross section of joint; (**c**–**e**) the microstructure of the laser-brazed specimen in A, B, C positions near the interface.

Nasiri et al. used nickel–coated steel [42,43], Al–12Si–coated steel [49], Sn–coated steel [45], Zn–coated steel [36] with AZ92 filler wire, to carry out a series of studies by diode laser brazed AZ31B Mg to steel sheet. As shown in Figure 11, dendritic AlNi IMC and α-Mg + Mg_2_Ni phases were formed along the interface at the bottom of the joint (zone A in Figure 11b), and only a dendritic AlNi phase was generated along the interface at the middle and top side of the joint (zone C in Figure 11b). The shear strength of the joint reached 96.8 MPa [42].

**Figure 12:**
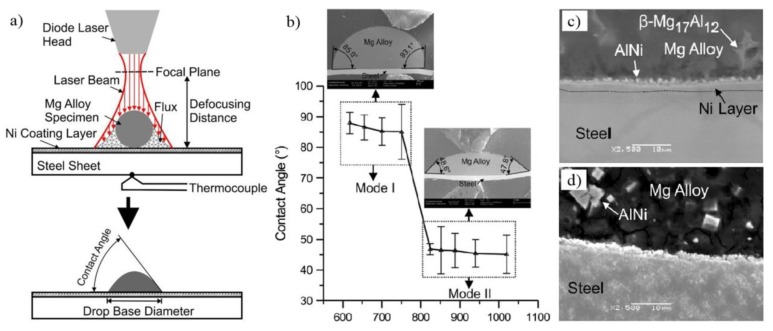
(**a**) Schematic representation of the wetting test; (**b**) the contact angle as a function of the peak temperature during wetting experiments; (**c**,**d**) the SEM micrographs of the Mg alloy-steel interface of the wetting sample at peak temperatures of 928 K and 1097 K [51].

Nasiri et al. also studied the wetting characterization by measuring the contact angles of AZ92 Mg alloy on Ni-electroplated steel as a function of temperature [51], as shown in Figure 12. Reactions between molten Mg and Ni led to a contact angle of about 86 deg in the temperature range of 891 K to 1023 K (Mode I (Mg-lNi-Mg_2_Ni-Ni-Fe)), and AlNi + Mg_2_Ni reaction products were produced between Mg and steel. Only 46 deg contact angle was detected in the temperature range of 1097 K to 1293 K (Mode II (Mg-AlNi-Fe)).

**Figure 13:**
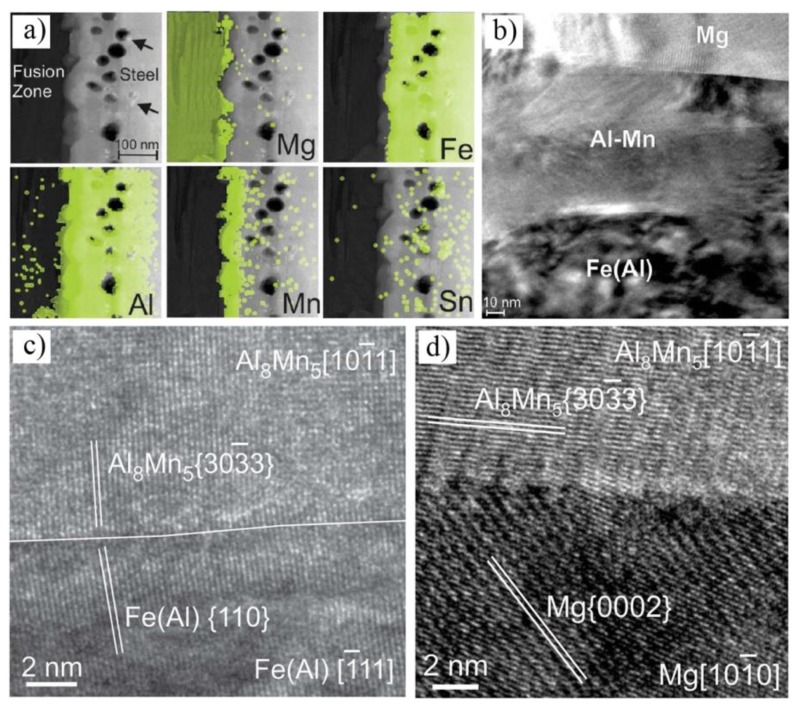
Laser welding of Mg/steel with Sn interlayer [45] (**a**) STEM-EDS concentration maps; (**b**) bright field TEM image of the Fe(Al)/Al_8_Mn_5_/Mg interfaces; (**c**,**d**) HR-TEM image of Fe(Al)/Al_8_Mn_5_ and Al_8_Mn_5_/Mg interface.

The interfacial microstructure of laser brazed AZ31B Mg to Sn-plated steel was also investigated [45]. Sn coating can protect steel from oxidation and promote liquid Mg’s direct reaction with steel fresh surface. A nano-scale layer is formed at the Mg/steel interface, composed of Fe(Al) solid solution and Al_8_(Mn, Fe)_5_, as shown in Figure 13. The Fe(Al)-Al_8_Mn_5_ interface showed that a crystallographic orientation relationship, $\left[10\bar{1}1\right]$_Al8Mn5_//$\left[\bar{1}11\right]$_Fe(Al)_, {110}_Fe(Al)_ was 4.2 deg from $\left\{30\bar{3}3\right\}$_Al8Mn5_ with 5.2% interplanar mismatch. The Al_8_Mn_5_-Mg interface showed a poor crystallographic matching between Al_8_Mn_5_ and α-Mg, $\left[10\bar{1}1\right]$_Al8Mn5_//$\left[10\bar{1}0\right]$_Mg_, $\left\{30\bar{3}3\right\}$_Al8Mn5_—within 47.4 deg of the {0002}_Mg_ with 16.8% interplanar mismatch—as shown in Figure 13b,c. All specimens were fractured in the steel base metal, and interfacial tensile shear strength exhibited more than 27.5 MPa.

**Figure 14:**
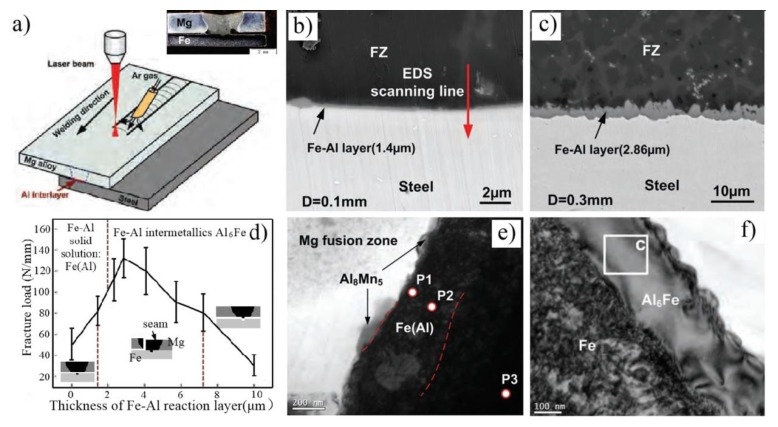
Laser-welding process of Mg to steel with the Al interlayer [44] (**a**) schematic diagram; (**b**,**c**) microstructure morphologies of interfacial reaction layers with 0.1 and 0.3 mm Al interlayer; (**d**) fracture load with a function of the thickness of the reaction layer; (**e**,**f**) TEM micrograph for reaction layer below/above 2 μm.

Tan et al. [44] investigated the influence of Al interlayer thickness on laser welding of Mg/Steel, as shown in Figure 14. The interfacial metallurgical reaction was improved after the Al interlayer melted. Two different reaction layers were formed and distinguished between them when the thickness of the reaction layer was below 2 μm. Fe(Al) solid solution and Al_8_(Mn, Fe)_5_ was formed at the Mg/steel interface and exhibited weak strength when the thickness of reaction layer was below 2 μm. The reaction layer was changed to Al_6_Fe IMC when the reaction layer varied between 2.3 μm and 5.7 μm, and the maximum value of the shear load reached 133 N/mm (40.3% steel base metal). Cracks were detected at the Mg/steel interface, which resulted in the joint strength sharply reducing with increase of the thickness of the reaction layer. However, Nasiri’s work indicated that the interfacial tensile shear strength could exhibit excellent performance with the composition of Fe(Al) solid solution and Al_8_(Mn, Fe)_5_ IMC at the Mg/steel interface [45]. It might be the continuity of the Al_8_(Mn, Fe)_5_ IMC that affects the performance of the joint.

**Figure 15:**
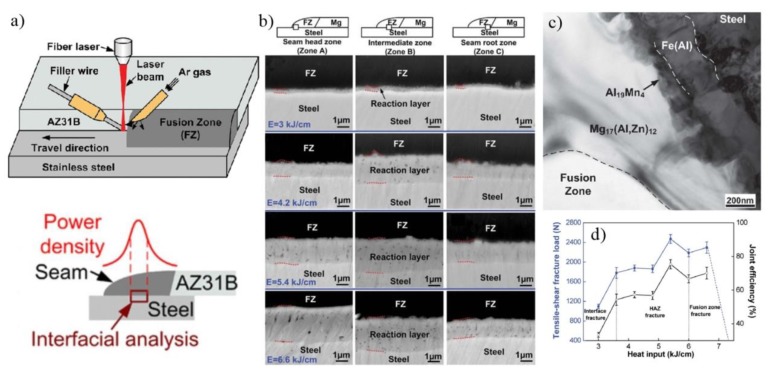
Laser welding-brazing with filler wire [47] (**a**) schematic diagram of the welding process; (**b**) interfacial microstructure at different heat inputs; (**c**) TEM bright field image at 5.4 kJ/cm heat input; (**d**) tensile-shear fracture load at different heat inputs.

Laser welding-brazing AZ31B Mg alloy to 201 stainless steel sheet with a AZ31 Mg alloy filler wire is shown in Figure 15 [47]. The reaction layer has obvious changes with the increase of heat input, as shown in Figure 15b. The tensile test results indicate that the maximum results value was 2472 N (75.4% Mg base metal) at 5.4 kJ/cm heat input. Three different fracture modes were observed: Interfacial failure at a lower heat input, fusion zone fracture at a higher heat input, and Mg base metal HAZ fracture at a suitable heat input. Figure 15c shows a bright field image, which was taken at the Mg/steel interface at 5.4 kJ/cm heat input. A nanoscale and continuous layer was detected near the Mg/steel interface, which was composed of three layer: Mg_17_(Al, Zn)_12_, Al_19_Mn_4_ and Fe(Al) solid solution.

### 3.2. Arc Welding-Brazing

#### 3.2.1. Metal Inert-Gas Welding-Brazing

**Figure 16:**
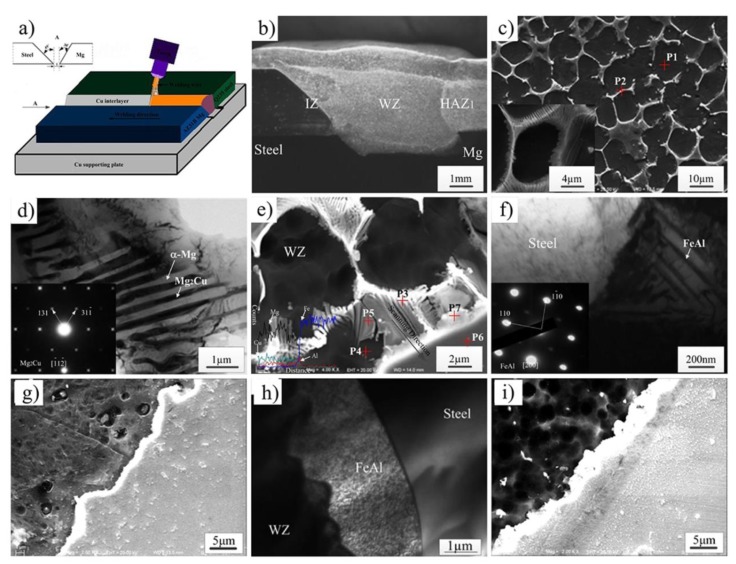
MIG welding process [53,54,55] (**a**) schematic diagram for Cu interlayer added; (**b**) cross-section of joint with 0.1 mm Cu; (**c**–**f**) microstructure of weld seam, eutectic structure, interfacial microstructure and reaction layer close to steel in (**b**), respectively; (**g**) interfacial microstructure for AZ31 filler wire added; (**h**) Mg/steel interface TEM image near in (**g**); (**i**) interfacial microstructure for AZ31 filler wire added.

The microstructure characteristics and tensile test results of AZ31 Mg/Q235 Steel dissimilar joints by MIG welding with a Cu interlayer are shown in Figure 16a [52,53]. The results show that the tensile strength was improved by the addition of a Cu interlayer, due to the improvement of wettability of Mg alloy on steel and the formation of eutectic structures (α-g + Mg_2_Cu) and Mg-Al-Cu ternary phases. The IMC Mg_2_Cu with a rod-like structure was generated in the weld zone and Mg/steel interfacial zone. The reaction layer was FeAl IMC, as shown in Figure 16f. The maximum tensile strength of joint reached 190 MPa with 0.15 mm Cu interlayer added (79% Mg alloy base metal), and the joint failed at the fusion zone and the Mg/steel interface together. The effect of Al content in Mg alloy filler wire on the microstructure and mechanical properties of the joint was also investigated. Figure 16g shows the Mg/steel joint, whose tensile strength could reach 184.2 MPa with the AZ31 filler metal added; its reaction layer was composed of FeAl IMC [54]. Joint strength could reach 192 MPa (80% AZ31 Mg alloy) with the AZ61 filler metal added [55].

Based on the above research, what they had in common was that the FeAl interface layer was obtained at Mg/steel interface and the joints exhibited better performance under the condition of adding a Cu interlayer or filler metal with different Al content. Liu et al. [56] also found that similar characteristics of joint strength with AZ31 filler metal added (171–174 MPa) was lower than the AZ61 filler metal added (201 MPa–83.8% Mg alloy base metal). The Mg/steel interface reaction layer was composed of double-layer structures, which were AlFe_3_ IMC layer and the Mg(Fe, Al)O_4_ + Mg_3.1_Al_0.9_ layer.

#### 3.2.2. Cold Metal Transfer Welding-Brazing

The cold metal transfer (CMT) welding process was based on common gas metal arc welding (MIG/MAG) plus the unique wire back pump technology (which allowed the wire to back pump in the event of a short circuit), reducing the heat input during welding.

**Figure 17:**
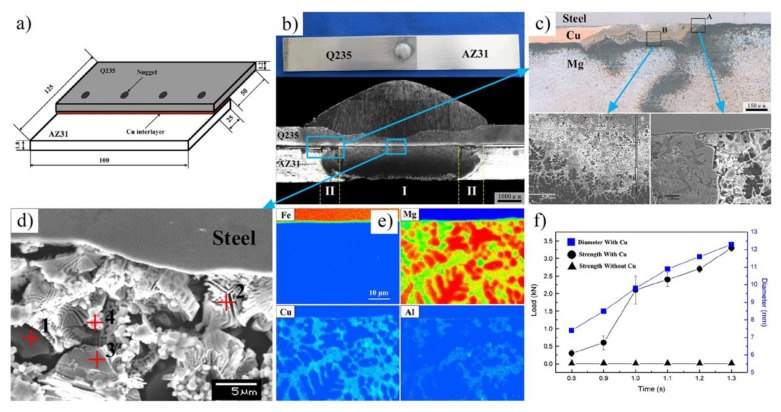
CMT spot welding with Cu interlayer [57] (**a**) schematic of the welding arrangement; (**b**) nugget surface and cross-section photograph; (**c**) microstructures of the interface at the nugget edge; (**d**) microstructures of the interface at the center of the nugget; (**e**) interfacial elemental distribution analysis at the center of the nugget; (**f**) relation between tensile shear strength and welding time.

Ren et al. [57] developed CMT spot joining AZ31 Mg alloy to Q235 steel with a Cu interlayer, as shown in Figure 17. There were two different bonding mechanisms at the Mg/steel interface: brazing at the nugget edge and welding-brazing at the center of the nugget. Composite reaction layers were composed of Al_3_Cu_4_Fe_3_ and Fe_4_Cu_3_ IMCs at the center of the nugget. Dissimilar AZ31 Mg alloy/Q235 steel joint could not be bonded by CMT spot welding without the Cu interlayer. The Cu interlayer played a bridging role in improving the weldability of Mg on a steel surface and promoting an interfacial metallurgical reaction.

The effects of Zn coating on Mg/steel lap-joints produced by the CMT method was studied [58,59]. The Mg/galvanized steel joint exhibited better spreadability and wettability than the Mg/bare steel joint. Two interlayers ((α-Mg + MgZn eutectic) + Fe-Al IMC) were formed at the Mg/galvanized steel interface, and Fe-Al IMC was formed at the Mg/bare steel interface. However, the maximum tensile load of Mg/bare steel joint (258 N/mm) was higher than that of the Mg/galvanized steel joint (224 N/mm). For the Mg/galvanized steel joint and the Mg/bare steel joint, the welding speeds were 5 mm/s, and wire feeding speed was 7.5 m/min and 10 m/min, respectively. The reason for the Mg/galvanized steel joint obtaining relatively low mechanical properties was that the bonding strength between the eutectic and the Fe-Al IMC at Mg/galvanized steel interface was lower than a thinner layer formed at the Mg/bare steel interface. Besides, CMT plug welding technology was also adopted to bond Mg AZ31 and hot-dipped galvanized mild steel, and Zn coating was the key to achieving a sound joint [60,61].

**Figure 18:**
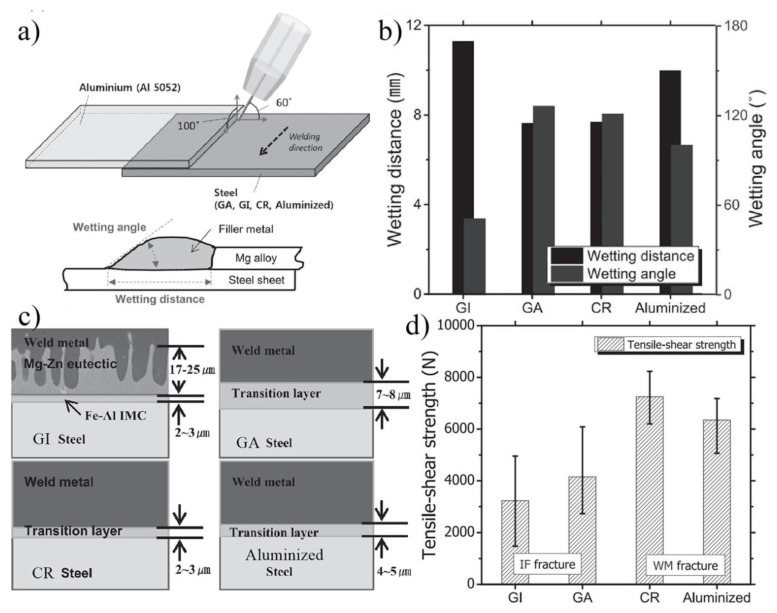
CMT lapped Mg-to-steel sheet welding process [62] (**a**) schematic diagram; (**b**) measured wetting angle and distance; (**c**) schematic of the transition layer; (**d**) tensile shear strength of the four types of brazed joints.

Kang et al. also investigated the effect of four different coatings on the properties of AZ31 Mg alloy/steel joints [62]. As shown in Figure 18, different coatings on the steel surface exhibited different wettability of Mg on steel. Wire feed speed also played a role in wettability [63]. The wettability of AZ31-galvanized (GI) steel joint and AZ31-aluminized (Aluminized) steel joint was better than AZ31-cold rolled bare (CR) steel joint and AZ31-galva-annealed (GA) steel joint. However, the bonding strength was not in accordance with the wettability of Mg on steel. The AZ31-CR and Aluminized steel joint exhibited higher bonding strength because Fe-Al phases were formed at the Mg/steel interface. The Mg-Zn-Al ternary phase was often detected at the Mg/steel interface of the AZ31-GI and AZ31-GA steel joints, which restricted the improvement of the joint performance. The reaction layer that was composed of the Mg-Zn eutectic and Fe-Al phases exhibited poor bonding strength because of the weak bonding between the Mg-Zn eutectic and the Fe-Al phases. In addition to the type of interface layer, the shape of the interface layer had a great influence on the mechanical properties of the joint. Chen et al. [64] found that the AZ31 Mg/Zn-coated DP600 steel spot joint strength (180 MPa) with a compact plate-like intermetallic Al_2_Fe layer was higher than the joint with an island-type Al_2_Fe interlayer.

### 3.3. Hybrid Heat Source Welding-Brazing

**Figure 19:**
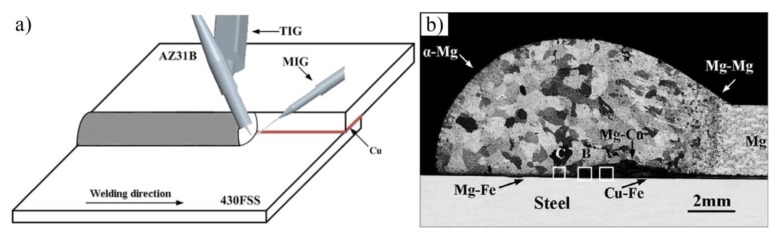
TIG-MIG hybrid welding with Cu interlayer [65] (**a**) schematic diagram; (**b**) cross-section photograph of the joints with 0.1 mm Cu interlayer.

TIG-MIG hybrid joining AZ31B Mg alloys to 430 ferritic stainless steel with a Cu interlayer is shown in Figure 19 [65]. The whole joint can be divided into three regions: Remaining region, full melting region, and partial melting region. The partial melting region is a major factor affecting bonding mechanism. A brazed Mg-Cu to steel joint was formed with the 0.02 mm Cu interlayer, and the IMCs transition layer was formed with the 0.1 mm Cu interlayer. The maximum shear strength of the joints with added 0.02 mm and 0.1 mm Cu interlayer was 57 MPa and 84 MPa, respectively. The tensile-shear strength was improved by 47%.

**Figure 20:**
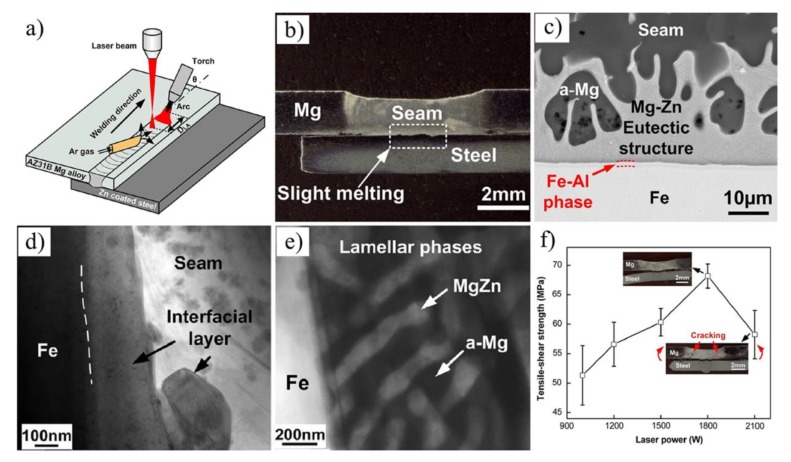
Mg/galvanized steel Laser-TIG hybrid welding process [66] (**a**) schematic diagram; (**b**) cross-section photograph; (**c**) interfacial microstructure morphologies at 1200 W; (**d**,**e**) bright field micrograph taken at the interface and the seam; (**f**) tensile-shear strength versus laser power.

Dissimilar metal of AZ31B Mg alloys and Zn coated DP980 steel was successfully joined by laser-TIG hybrid heat source welding-brazing [66], as showed in Figure 20a. The presence of Zn coating could prevent oxide formation at the Mg/steel interface. There were two bonding mechanisms: Fe/α-Mg + MgZn eutectic structure/Mg interfacial structure and the Fe/Fe_3_Al/Mg interfacial structure. At times, the Al_6_Mn phase is formed adjacent to the Fe_3_Al IMC. The maximum tensile-shear strength obtained was 68 MPa (52.3% Mg alloy base metal) at 1800 W laser power.

## 4. Fusion Welding

The laser-arc hybrid heat source welding technology is a new technology that combines a laser heat source and an arc heat source, thus offering the advantages of controllable energy density, high efficiency, and strong adaptability. It is now widely applied to same species and dissimilar metal welding. The fusion welding method ensures that the whole interface is always in a high temperature zone and provides a new interfacial reaction mechanism for welding of immiscible Mg/steel. This idea is completely different from that of welding-brazing and solid-state welding processes. However, there are challenges in obtaining high interfacial temperatures. The higher the interfacial temperature, the harder the welding formation is. It is impossible for Fe and Mg to coexist in a liquid state. As Mg and steel melt at the same time, Mg near the melted steel will be directly vaporized. Laser-TIG hybrid welding addresses this challenge by offering temperature gradient distribution [67,68]. High-energy density laser pulse can be applied to the border of Mg alloy and steel to increase the interfacial reaction temperature, while a low-energy density arc acts on the Mg alloy to reduce the burning loss, which benefits the welding formation. Existing studies show that an alloy element is the key to promoting the formation of an interface layer for immiscible Mg/steel joints; for instance, AZ series Mg alloy filler wire, Al, Ni, Cu, and Cu-Zn interlayer.

### 4.1. Laser-Arc Hybrid Heat Source Lap Welding

AZ31B Mg alloy and 304 steel is directly welded by laser-TIG welding in a lap joint without any interlayer or filler wire [69]. Metallic oxides produced at the Mg/Fe interface were the reason for the poor mechanical properties of the weld joints.

**Figure 21:**
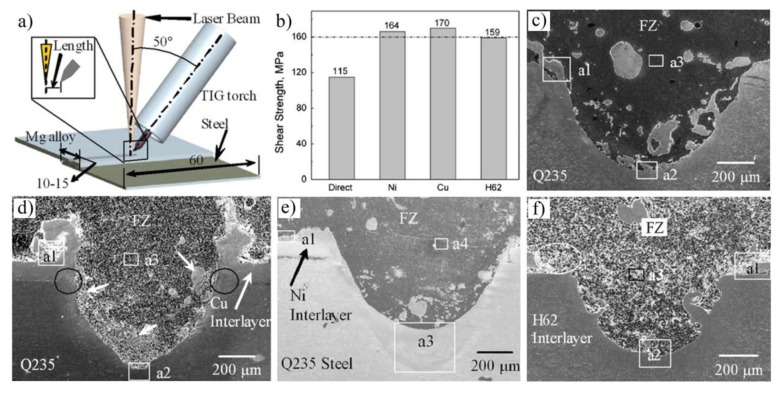
Laser-TIG lap welding with different interlayers [70] (**a**) schematic diagram of welding; (**b**) strength comparison of various interlayer-added joints; (**c**–**f**) microstructures of Mg/steel joints with no Cu, Ni, Cu-Zn interlayer.

In order to minimize metallic oxides at the Mg/Fe interface, Liu et al. conducted extensive research on laser-TIG lap fusion welding with an interlayer such as Ni [68,70,71,72,73,74,75,76,77], Cu [70,71,72,74,75,78], Sn [72,79], and Cu–Zn [70,72] interlayer. The shear strength of a joint without any interlayers added was much lower than that with an interlayer. Interlayer selection should follow the principle of elevating its wettability on steel and avoiding massive production of brittle intermetallics in the joint. Morphology of different interlayer joints are shown in Figure 21. Fe-Ni solid solutions were formed close to steel and Mg_2_Ni IMC close to the Mg alloy with an Ni interlayer added. Mg_2_Cu IMC with rod-like structures in the joint and an equiaxed structure at the interface were found with a Cu interlayer added. Mg_2_Sn IMC with a dendritic structure distributed was identified in the grain boundaries of the Mg alloy with an Sn interlayer added [79]. A comparison of the tensile-shear properties of joints with different interlayers can be seen in Figure 21b.

### 4.2. Laser-Arc Hybrid Heat Source Butt Welding

**Figure 22:**
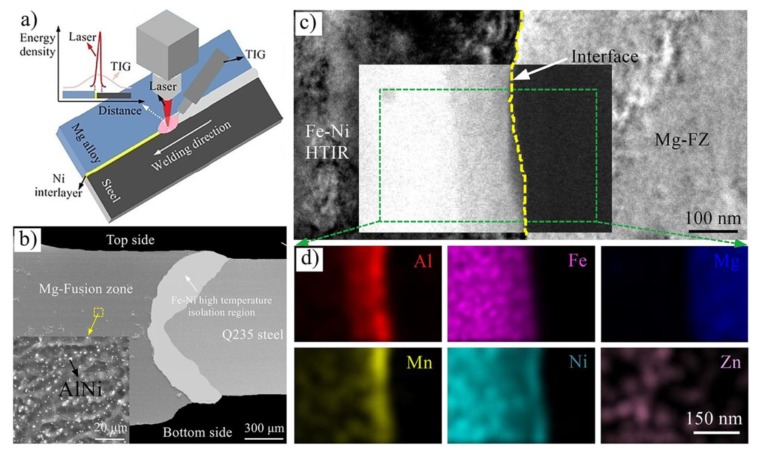
Laser-TIG double side welding process with Ni interlayer [81] (**a**) schematic representation of the laser-TIG process; (**b**) Cross section of joint; (**c**) TEM bright field image and HAADF-STEM image near the interface; (**d**) elemental distribution mapped with STEM near the interface marked in (**c**).

The effect of gradient thermal distribution on the microstructure and property of an Mg/steel butt joint was investigated [67]. The maximum tensile strength value was 203 MPa with laser offset to steel 0.2 mm. Interfacial metallurgical reaction occurred at the Mg/steel interface alloy and formed a Cu-Mg-Zn IMC and Fe-based (Fe, Cu, Al) solid solution. Besides, the Ni interlayer was also adopted to bond to an unequal thickness Mg/steel butt-welded plate [80,81], as shown in Figure 22. Laser-TIG double-sided welding was adopted to melt the Ni interlayer, which required more heat input than with a Cu-Zn interlayer. Uniformly distributed fine particles (AlNi) were formed in the fusion zone, which was conducive to improving weld strength. The maximum tensile strength value was 232 MPa with laser offset to steel 0.1 mm. The transition zone between the weld seam and the steel base metal was composed of (Al, Mg) (Ni, Mn, Fe) intermetallic-based solid solutions (150 nm thickness) and α-(Fe, Ni) solid solution (60–500 μm thickness). The interplanar of (110)_(Al, Mg) (Ni, Mn, Fe)_‖(0002)_Mg_ and interatomic of $\left[\bar{1}11\right]$_(Al, Mg) (Ni, Mn, Fe)_ and $\left[11\bar{2}0\right]$_Mg_ misfits for the (Al, Mg) (Ni, Mn, Fe)/Mg system were reduced by 42.2% and 41.2%.

**Figure 23:**
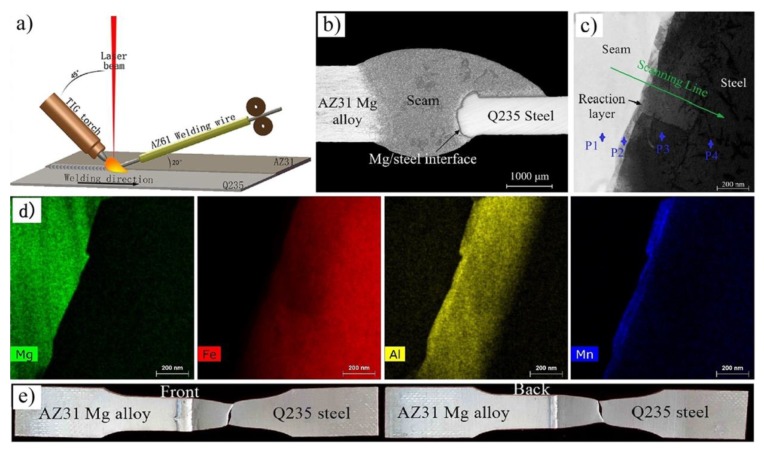
Laser-TIG hybrid welding with filler wire [83] (**a**) schematic diagram of welding; (**b**) cross-section appearance for 850 W; (**c**) STEM micrograph taken from the interface of Mg/steel joint; (**d**) elemental distribution of Mg, Fe, Al, Mn; (**e**) fracture location of joint.

The AZ61 filler wire was also used in butt fusion welding of AZ31B Mg alloy and Q235 steel [82,83]. A reaction layer is formed at the welding interface enriched with Al and Mn elements, as shown in Figure 23. The morphology of Mg/steel joint and thickness of the transition layer were the key factors influencing the properties and fracture behavior of the welded joint. The Mg/steel interface layer was composed of HT-Al_11_(Mn, Fe)_4_ IMC (50 nm thickness) and α-Fe(Al) solid solution (350 nm thickness). The misfit was 2.69% along (011)_α_-_Fe(Al)_‖(060)_HT_-_Al11(Mn, Fe)4_ planes and 1.67% along $\left[1\bar{1}0\right]$_α_-_Fe(Al_)‖[100]_HT_-_Al11(Mn, Fe)4_ directions with theoretical calculations. The misfit was 3.50% along $\left(01\bar{1}2\right)$_Mg_‖(060)_HT_-_Al11(Mn, Fe)4_ planes and 17.68% along $\left[10\bar{1}0\right]$_Mg_‖[100]_HT_-_Al11(Mn, Fe)4_ directions with theoretical calculations. The tensile strength reached 238.1 MPa (94.5% of AZ31 base metal) with the weld reinforcement removed, and the joint failure approach at the Mg/steel interface.

## 5. Other Processes

### 5.1. Resistance Spot Welding

In resistance spot welding (RSW), a joint is welded under a certain pressure and current. When resistance heat is generated and applied to the interface of the workpiece, the materials are bonded.

**Figure 24:**
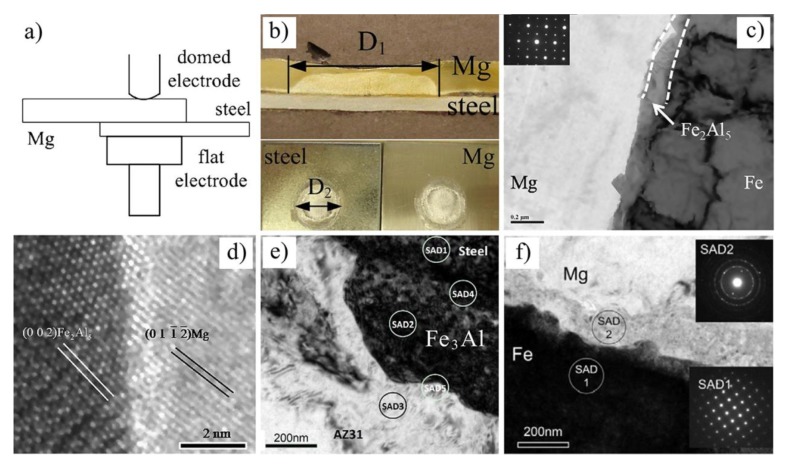
Mg/steel RSW process [87,89,90] (**a**) schematic diagram; (**b**) cross section of weld and fracture surface; (**c**) Mg/Fe_2_Al_5_/Fe interface structure; (**d**) HRTEM of Fe_2_Al_5_ and Mg; (**e**) bright field image of the interface IMC layer of AZ31/DP600 steel joint; (**f**) high magnification image of the Mg/IF steel interface.

Dissimilar metals of AZ31B Mg alloy and steel joints were successfully bonded by RSW [84,85,86,87,88,89,90], as shown in Figure 24. Liu et al. found that a precoated nanoscale Fe_2_Al_5_ transition layer matched well with Mg and steel lattices, and could help obtain a sound joint [89]. This is because well-matching lattice sites have low-energy interfaces, which boost the interface bonding strength. The study found that Fe_2_Al_5_ and Fe matched well with the OR of (002)_Fe2Al5_‖(110)_Fe_ planes and $\left[1\bar{1}0\right]$_Fe2Al5_‖$\left[1\bar{1}1\right]$_Fe_ direction, Fe_2_Al_5_ and Mg matched well with the OR of (002)_Fe2Al5_‖$\left(01\bar{1}\bar{2}\right)$_Mg_ planes and $\left[0\bar{1}0\right]$_Fe2Al5_‖[100]_Fe_ direction. Besides, they also found that an interfacial intermetallic transition layer was not the only measure of successful bonding of dissimilar Mg/steel metals. A Mg nano grain layer could also bond immiscible pure Mg-Fe dissimilar materials [87]. However, the weld strength of AZ31/DP600 joint (5.7 kN) with an Fe_3_Al interfacial layer was higher than a pure Mg/Fe joint (2.08 kN).

**Figure 25:**
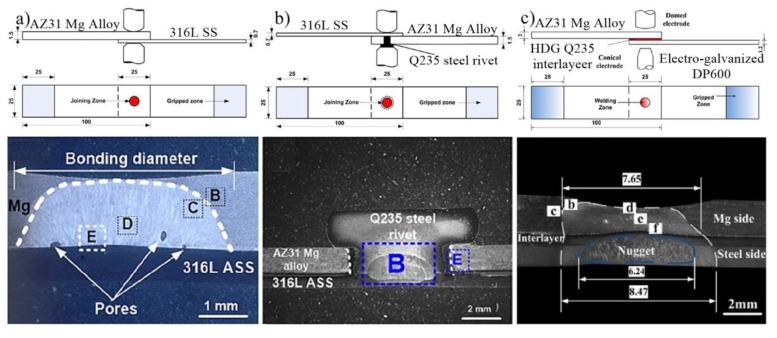
Mg/steel RSW process [84,85] (**a**) schematic diagram of the traditional RSW process and cross-section of the weld; (**b**) schematic diagram of the REW process and cross-section of the weld; (**c**) schematic diagram of RSW with Q235 interlayer process and cross-section of the weld.

Besides resistance element welding (REW) [84], RSW with a hot-dip galvanized Q235 steel interlayer [85] and RSW with a cover plate [86] were also adopted to bond Mg/steel dissimilar metals. As shown in Figure 25a, only a nugget was formed in the Mg alloy, which had a welding-brazing character for the AZ31 Mg alloy/316 L austenitic stainless steel joint. Two nuggets were formed during REW (Figure 25b): One near the Q235/316 L interface and the other in the Mg alloy close to the Mg/Q235 interface [84]. The peak load of the REW joints was 3.71 kN, which was higher than that of RSW joints (2.23 kN). Similarly, a high quality AZ31/DP600 joint was obtained by RSW with a hot-dip galvanized Q235 steel interlayer [85], as shown in Figure 25c. The maximum peak load (5.49 kN) was increased by 32.6%, when compared to a traditional RSW joint (4.14 kN).

**Figure 26:**
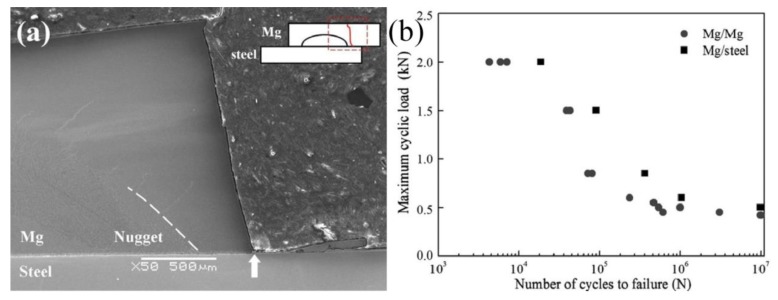
Mg/steel RSW [88] (**a**) fatigue fractures at a maximum load of 2.0 kN; (**b**) fatigue properties.

AZ31B Mg alloy/hot-dip galvanized HSLA steel dissimilar metals were successfully welded by resistance spot welding [88]. The fatigue performance of Mg/steel welds was almost the same as that of Mg/Mg welds, as shown in Figure 26. Mg/steel and Mg/Mg welds were fractured in the thickness direction of Mg alloy under stress-controlled cyclic loading. Zinc coating enters the molten pool to prevent liquid metal embrittlement, which results in fatigue fracture appearing in the thickness direction of the Mg alloy.

### 5.2. Ultrasonic Spot Welding

**Figure 27:**
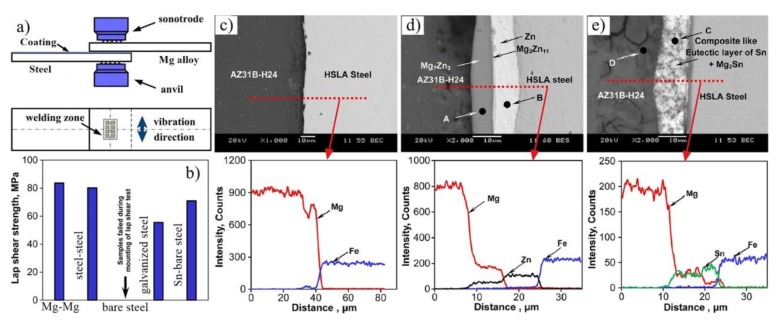
Mg/steel USW process [91] (**a**) schematic diagram of USW process; (**b**) Lap shear strength for different coated steel; (**c**) Mg–to–bare steel; (**d**) Mg–to–galvanized steel and (**e**) Mg–to–bare steel with Sn interlayer

Mg/steel dissimilar metals such as Mg-to-galvanized steel [91,92,93,94,95], Mg-to-bare steel with Sn interlayer [91,96], and Mg-to-galvanized steel with adhesives [97,98] were successfully bonded by ultrasonic spot welding (USW). The effects of different steel surface coatings on the microstructure and properties of USW Mg/steel joints are shown in Figure 27 [91]. Weak interface bonding strength was obtained for USW Mg-to-bare steel. For Mg-to-bare steel with a Sn interlayer joint, the transition layer was composed of Sn and a Mg_2_Sn eutectic structure [91,96]. Mg_7_Zn_3_ and Mg_2_Zn_11_ were presented in the interface of the Mg-to-galvanized steel joint [91], and sometimes detected as MgZn_2_ IMC together [92]. The shear strength of the Mg/Sn coated steel joint (71 MPa) was higher for Mg/bare steel (samples failed during mounting of the lap shear test) and Mg/Zn coated steel joint (56 MPa).

## 6. Summary

An Mg/steel dissimilar metal hybrid welding structure has both strength requirements and light weight advantages, and offers broad application prospects for the future. Therefore, scholars have been using different welding techniques to carry out systematic research. The key issues for Mg/steel dissimilar metal joints are the large difference in melting point, no Mg-Fe IMCs, and little solid solubility in an Mg-Fe system.

Solid-phase welding (friction stir welding and diffusion welding) can effectively solve the problem of large differences in melting points of Mg/steel dissimilar metals and avoid welding defects in fusion welding. In order to promote the metallurgical bonding interface of Mg and steel, a large number of scholars have adopted the welding-brazing method. A heat source is applied to the Mg alloy (base metal or filler material), melting the Mg alloy by utilizing the difference in melting point between the Mg alloy and steel. The use of a cladding metal or an interlayer metal could effectively promote infiltration of the alloy elements into the solid steel base metal, and achieve a metallurgical bonding interface during welding-brazing, such as laser welding-brazing, arc welding-brazing, and hybrid heat source welding-brazing. Besides, Mg/steel fusion welding is also a promising technique that could promote interfacial metallurgical reactions and obtain more stable intermetallic compounds (IMC). The laser-TIG hybrid welding technique is a typical method that deals with the issue of large differences in melting and boil points between Mg and Fe. The high-energy density laser could increase interfacial temperature and promote interfacial reaction, and the low energy density arc could reduce evaporation of the Mg alloy.

Although the bonding of Mg alloy and steel has made great progress, there are several problems that need to be solved. The key factors that affect the performance of the joint are the thickness, continuity, and type of the interface layer. Few studies have focused on the design of the interface layer type, and the effect of different types of interface layers on the bonding of Mg/steel dissimilar metals. The Mg/steel interface bonding mechanism is still not clear. Although an edge-to-edge matching model was used to predict orientation relationships (OR) of Mg/IMC and IMC/steel and characterize bonding strength of interfaces, there are not enough matching theories to compare the interface strengthening mechanism of different interface layers. There are others aspects that also need to be further studied, such as fracture mechanism, corrosion mechanism, fatigue properties of Mg/steel joint, etc. Hence, there is still more research to be conducted on the bonding mechanism of magnesium/steel joints.

## Figures and Tables

**Table 1:** The physical and chemical properties of Mg and Fe [8].

Properties	Mg	Fe
Crystal structure	hexagonal close-acked	body-centered cubic and face-centered cubic
Atomic radius (pm)	160	126
Melting point (K)	923	1811
Boiling point (K)	1363	3134
Thermal conductivity (W/(m∙K))	156	80.4
Young’s modulus (GPa)	45	211
Thermal expansion (μm/(m·K)) (at 25 °C)	24.8	11.8
Density (g/cm^3^)	1.738	7.874
Heat of vaporization (kJ/mol)	128	340
Heat of fusion (kJ/mol)	8.48	13.81
